# Peripheral neuromodulation in spasticity-plus syndrome: effects of pulsed radiofrequency on tonic-painful disorders in multiple sclerosis

**DOI:** 10.3389/fneur.2025.1634960

**Published:** 2025-09-22

**Authors:** Luigi Di Lorenzo, Carmine D’Avanzo

**Affiliations:** Mediterranean Neurological Institute Neuromed (IRCCS), Pozzilli, Italy

**Keywords:** spasticity plus syndrome, neuromodulation, painful spasticity, pulsed radiofrequency, multiple sclerosis

## Abstract

**Introduction:**

Spasticity-Plus Syndrome (SPS) in multiple sclerosis (MS) represents a cluster of symptoms including spasticity, neuropathic pain, spasms, and bladder dysfunction. These manifestations may worsen after trauma or surgery. Pulsed radiofrequency (PRF) offers a minimally invasive neuromodulation strategy that could complement standard treatments.

**Methods:**

We report the case of a 56-year-old woman with secondary progressive MS (EDSS 6.5) who developed SPS after hip arthroplasty. Despite multiple pharmacological therapies (baclofen, opioids, nabiximols), symptom control remained poor. Two diagnostic nerve blocks were performed, followed by PRF of the femoral and obturator articular branches. Outcomes were monitored using patient-reported measures, the Modified Ashworth Scale, and the Numerical Rating Scale.

**Results:**

PRF induced a 50–60% reduction in pain and a marked decrease in spasms, with partial improvements in sleep and quality of life. The patient rated PRF as superior to all prior treatments. Benefits were sustained for several months, supporting repeat PRF and adjunctive nerve blocks during follow-up.

**Discussion:**

This case illustrates the role of PRF in SPS management when pharmacological options are insufficient or poorly tolerated. PRF provides safe, repeatable peripheral neuromodulation without neuro-destructive effects, enabling multimodal, patient-centered care. Although based on a single case, these findings support the clinical value of the SPS construct and suggest PRF as a promising complementary strategy in MS-related disability.

## Introduction

Spasticity is a common manifestation of multiple sclerosis (MS), classically defined as a velocity-dependent increase in muscle tone. Spasticity and muscle weakness are the most disabling symptoms in people with Multiple Sclerosis, frequently affecting lower limbs and causing motor impairments, fatigue and increased risk of falls (1). However, in clinical practice, spasticity rarely presents as an isolated symptom. It is often accompanied by muscle spasms, neuropathic pain, sleep disruption, bladder dysfunction, and fatigue— collectively referred to as the Spasticity-Plus Syndrome (SPS). This model, initially proposed by Ramió and further developed by Centonze and colleagues (2, 3), reflects a more comprehensive view of MS symptomatology, emphasizing the shared neurophysiological mechanisms underlying these manifestations. The pathophysiological substrate of SPS is thought to involve demyelination of small-diameter axons, which increases vulnerability to conduction block and ephaptic transmission. This phenomenon may explain the co-occurrence of irritative (e.g., spasms, pain) and deficit (e.g., weakness, fatigue) symptoms. The concept of SPS encourages an integrated therapeutic strategy targeting dysfunctional neural circuits, potentially improving multiple symptoms simultaneously and minimizing reliance on polypharmacy (1–3). Case Report This report presents a case of SPS triggered and exacerbated by an orthopedic event in a patient with secondary progressive MS (SPMS). It explores the therapeutic impact of peripheral neuromodulation using pulsed radiofrequency (PRF), a minimally invasive technique that modulates sensory input at the segmental level without causing neural destruction (4).

## Case report

A 56-year-old female patient with a diagnosis of secondary progressive multiple sclerosis (SPMS) and an Expanded Disability Status Scale (EDSS) score of 6.5 presented to our attention. The patient had significant pre-existing weakness and spasticity of the right lower limb (EDSS 6.5), but was still ambulatory indoors with bilateral support before the fall. Although the patient had an EDSS score of 6.5, she was still ambulatory indoors using bilateral support. The EDSS includes structured functional assessments, and in this case, the pyramidal and sensory system scores reflected a significant but stable impairment of the right lower limb prior to the fall The diagnosis of Spasticity-Plus Syndrome (SPS) was made based on the presence of a characteristic cluster of symptoms—including increased muscle tone, painful spasms, neuropathic pain, ad neurogenic bladder dysfunction—following the criteria described by Centonze et al. and Fernández et al. (2, 3). A structured clinical evaluation was conducted, including the Modified Ashworth Scale for spasticity, the Numerical Rating Scale for pain, and targeted questions regarding bladder function and sleep quality.

Fernández et al. recently developed a practical algorithm—the IDSPS tool—to identify Spasticity-Plus Syndrome (SPS) in multiple sclerosis patients, based on a conjoint analysis of expert-rated symptom profiles (5). The tool estimates the probability of SPS by weighting clinical features such as spasticity, spasms, and bladder dysfunction. In our study, we clinically assessed this and other patients with MS using the conceptual framework proposed in that work. As in the routine practice of our MS unit, we adopted a syndromic approach without formally applying the IDSPS algorithm.

Nonetheless, the tool informed our clinical reasoning and supports the need for early recognition of complex symptom clusters. Approximately 10 months earlier, the patient had sustained a right fem- oral neck fracture after attempting to rise unassisted. The traumatic event was surgically managed with a total hip arthroplasty. Beginning in the first postoperative month, the patient reported a progressive increase in pain localized to the right hip, accompanied by worsening spasticity of the adductor muscles and the onset of extensor spasms involving the ipsilateral hamstrings, rectus femoris, and vastus medialis. The escalation of pain was associated with an aggravation of spasticity, deterioration in sleep quality and mood, and impaired management of neurogenic bladder function.

Postoperative imaging, including standard hip radiographs, confirmed proper alignment of the prosthesis with no evidence of loosening, dislocation, or other mechanical complications. Therefore, surgical revision was not indicated. The overall clinical picture was consistent with a manifestation of Spasticity-Plus Syndrome. Two months after surgery, treatment was initiated with nabiximols (Sativex®), an oromucosal spray formulation of THC: CBD approved for the treatment of spasticity in MS (6, 7). However, the patient reported minimal benefit. Although nabiximols (Sativex®) are considered effective in cannabinoid-responsive symptoms and have contributed to the formulation of the Spasticity-Plus Syndrome (SPS) concept, the patient experienced only minimal improvement (7, 8). This may reflect the predominance of peripheral nociceptive inputs and segmental sensitization mechanisms less amenable to central cannabinoid modulation. Additionally, individual variability in endocannabinoid system responsiveness could play a role. These findings reinforce the hypothesis that SPS includes subtypes with differing pathophysiological profiles, some of which may benefit more from peripheral neuromodulation (7) than from systemic pharmacotherapy (1–8). Before considering neuromodulation, the patient was treated with a wide range of pharmacologic therapies. These included paracetamol and NSAIDs, pregabalin up to 300 mg/day, duloxetine 60 mg/day, benzodiazepines (e.g., diazepam), transdermal buprenorphine (up to 25 mcg/h), and transdermal fentanyl (up to 25 mcg/h) (9). For breakthrough pain, fentanyl lollipops were also used. Although MS is a highly prevalent autoimmune disorder. Opioid peptides and their receptors are intimately involved in regulating various aspects of immune function, nociceptive processing, and affective states. Dysregulation of the opioid system may be an important mechanism to help explain the pathophysiology of MS, as well as the pathological pain and disordered mood commonly observed in this disease. Therefore, it is of interest to further investigate and consider the opioid system as a potentially at- tractive therapeutic target for MS and its symptoms (9).

Despite this extensive pharmacological regimen, clinical improvement was minimal and transient, and side effects such as sedation and reduced alertness limited therapeutic adherence and participation in rehabilitation. These factors supported the decision to initiate a neuromodulatory strategy with targeted PRF. At the sixth postoperative month, to assess the peripheral and articular contribution to pain and spasticity, two diagnostic nerve blocks were performed: one targeting the trochanteric branches of the femoral nerve and the other aimed at the superior gluteal nerves. Both procedures resulted in a temporary yet significant reduction in pain and spasms. In light of the favorable response, definitive treatment was carried out with pulsed radiofrequency (PRF) of the same neural targets. Pulsed radiofrequency (PRF) treatment was performed using a standardized protocol with the following parameters: 2 Hz frequency, 20 ms pulse width, 120 s per target site, and a maximum electrode temperature of 42 °C. These settings are in accordance with established protocols for non-destructive neuromodulation of peripheral sensory nerves Interventions were performed by a physician certified in both Physical & Rehabilitation Medicine and Anesthesiology, under ultrasound guidance. The trochanteric branches of the femoral and obturator nerves were targeted following validated techniques in the literature. Femoral articular branches traverse the iliopsoas plane to innervate the anterolateral hip capsule; obturator branches supply the inferomedial capsule; the PENG block (10), involves in-plane needle insertion between the psoas tendon and pubic ramus, aiming at the plane of the iliopubic eminence to modulate articular branches of the femoral, obturator, and accessory obturator nerves. Several weeks after the initial PRF procedure, the patient reported a 50–60% reduction in pain compared to baseline, along with a marked decrease in the frequency and intensity of spasms and clonus. After PRF, the patient reported partial improvement in sleep and pain but no meaningful change in bladder symptoms, which remained stable. Although pain relief may influence autonomic symptoms indirectly, this was not observed in our case. In light of the suboptimal response to previous pharmacological management—including baclofen, buprenorphine, and nabiximols—the approximately 50–60% global improvement achieved with PRF was clinically meaningful and subjectively rated by the patient as superior. Despite the transient nature of the benefit, the positive outcome led to the implementation of a quarterly follow-up plan. This includes ipsilateral PENG block, transversus abdominis plane (TAP) block, and erector spinae plane block, all designed to reinforce pharmacological neuromodulation. These procedures also serve as a decision-making framework to re-evaluate the need for further PRF treatments in a personalized, multimodal therapeutic strategy.

## Discussion

The clinical case described represents a paradigmatic example of Spasticity-Plus Syndrome (SPS), in which an acute orthopedic event acted as a triggering or amplifying factor of a pre-existing cluster of symptoms, including spasticity, muscle spasms, and neuropathic pain. This case contributes to the clinical understanding of Spasticity-Plus Syndrome by exemplifying how a multifaceted symptom cluster—including spasticity, neuropathic pain, and bladder dysfunction—can emerge and worsen in the context of peripheral trauma in a patient with MS. Although this is a single-case report, it supports the application of the SPS framework in real-world clinical reasoning and personalized therapeutic planning, in line with the criteria proposed by Fernández et al. and Centonze et al. At the time of manuscript submission, the patient had completed 9 months of follow-up, during which she underwent quarterly scheduled interventions consisting of PENG, TAP, and erector spinae blocks. These procedures allowed for sustained symptom control following the initial PRF, although the analgesic and antispastic effects had gradually attenuated by the sixth month. A second PRF session targeting the PENG region and potentially the obturator nerve is currently planned. These longitudinal data support the notion that repeated peripheral neuromodulation may offer a feasible strategy for long-term management of SPS in selected MS patients. In this patient, hip prosthesis implantation likely increased both peripheral and central sensitization, contributing to the activation of hyperexcitable spinal circuits characteristic of SPS. This phenomenon can be explained by the pathophysiological theory proposed by Centonze et al., according to which the demyelination of small-diameter axons, susceptible to ephaptic transmission and conduction block, may lead to the overlap of irritative and deficit symptoms (2, 3). In this context, neuromodulation strategies are becoming increasingly relevant in the integrated treatment of SPS. Among the potential interventions, intrathecal baclofen therapy was also considered during the clinical course. However, after detailed explanation of the procedural aspects, the patient declined consent, expressing a preference for less invasive and more reversible approaches. This patient-centered decision contributed to the selection of peripheral neuromodulation, which offered a safer and well-tolerated alternative in this context. This choice was already mentioned in the manuscript and is now further clarified. Repetitive transcranial magnetic stimulation (rTMS) and transcranial direct current stimulation (tDCS) are non-invasive techniques that have demonstrated efficacy in experimental settings in modulating cortical plasticity and reducing spasticity symptoms in patients with MS and other central nervous system lesions (8, 11, 12). However, these approaches mainly act at the supraspinal and cortical levels and have not yet been fully integrated into current clinical practice, with relatively limited effectiveness on aberrant segmental transmission and on exaggerated spinal reflexes that contribute to spasms and clonus (1–3). In this scenario, the rationale for the use of pulsed radiofrequency (PRF) may therefore be proposed—a non-destructive peripheral neuromodulation technique that differs from continuous radiofrequency by its lack of neurodestructive effects and its ability to selectively modulate the activity of sensory nerves (4, 13–18). PRF operates by generating short high-frequency electrical impulses at low temperatures (≤42 °C), which result in an alteration of the neuronal microenvironment, reducing membrane excitability and interfering with nociceptive signal transmission without causing structural damage (14). At the segmental level, PRF has been associated with a reduction in abnormal ephaptic transmission, suppression of neuronal hyperexcitability, and modulation of ion channels responsible for the propagation of action potentials in sensory neurons (14). These effects are particularly useful in patients with MS, in whom demyelination renders axons more susceptible to crosstalk phenomena. Scientific evidence exists to support the assertion that pulsed radiofrequency (PRF) can modulate segmental neuronal excitability, reduce abnormal ephaptic transmission, and influence ion channels involved in the propagation of action potentials in sensory neurons. A study published in Pain Physician highlighted that PRF modulates various ion channels, including Na^+^/K^+^ ATPase, HCN, and P2X3, in addition to affecting neurotransmitters and postsynaptic receptors such as AMPA-R and GABA-B (17). All these effects contribute to the reduction of nociceptive transmission and the attenuation of neuropathic pain (16–20). These comprehensive analysis showed that PRF induces microscopic and biochemical changes in neurons and glial cells, regulating inflammatory responses and gene expression related to pain transmission (19). These effects include modulation of structures such as myelin, mitochondria, and ion channels, contributing to the antinociceptive effect of PRF (16, 17, 19, 21). Beyond nociceptive modulation, recent preclinical studies suggest that PRF may attenuate microglial activation and reduce pro-inflammatory cytokines such as TNF-*α* and IL-1β. These effects might influence the neuroinflammatory milieu typical of MS and indirectly support oligodendrocyte survival and remyelination (22). While evidence in demyelinating disease models remains preliminary, such findings raise the possibility that PRF could impact immune-neural interactions relevant to the pathogenesis of Spasticity-Plus Syndrome (5, 20). From a practical standpoint, PRF presents as an advantageous technique for several reasons: it allows for focal and selective action on sensory branches (such as the trochanteric branches of the femoral and obturator nerves), has an excellent safety profile compared to continuous radiofrequency (which can cause thermal nerve injury), is easily repeatable over time, and provides significant pain relief that facilitates active participation in functional rehabilitation (16–19). In the case described, the combination of diagnostic nerve blocks followed by targeted pulsed radiofrequency (PRF) of the articular branches of the femoral and obturator nerves resulted in a clinical benefit greater than that achieved with pharmacologic therapy or botulinum toxin alone. This outcome supports both the clinical validity of the Spasticity-Plus Syndrome (SPS) construct and the therapeutic expansion of peripheral neuromodulation strategies such as PRF within a personalized, multimodal framework. The therapeutic role of pulsed radiofrequency (PRF) in neuropathic pain syndromes is well supported in both clinical and experimental literature. Beyond the single case context, PRF has been recognized in international guidelines, including those of the International Neuromodulation Society (INS) and the Neuromodulation Appropriateness Consensus Committee (NACC), as a valid and evidence-based tool for managing chronic neuropathic pain. Its non-destructive, focal mechanism of action and favorable safety profile make it especially suitable for integration into multimodal pain management protocols, particularly when pharmacological strategies have failed or caused intolerable side effects. Finally, this report is limited by the use of predominantly subjective outcome measures, including patient-reported improvements in pain, spasms, and sleep. While some clinical scales such as the Modified Ashworth Scale and Numerical Rating Scale were used at baseline, they were not consistently applied at each follow-up. Electrophysiological assessments, such as EMG or H-reflex, were not conducted. Future studies should incorporate standardized quantitative tools and objective neurophysiological indicators to strengthen evidence on the efficacy of PRF in Spasticity-Plus Syndrome.

**Figure 1:**
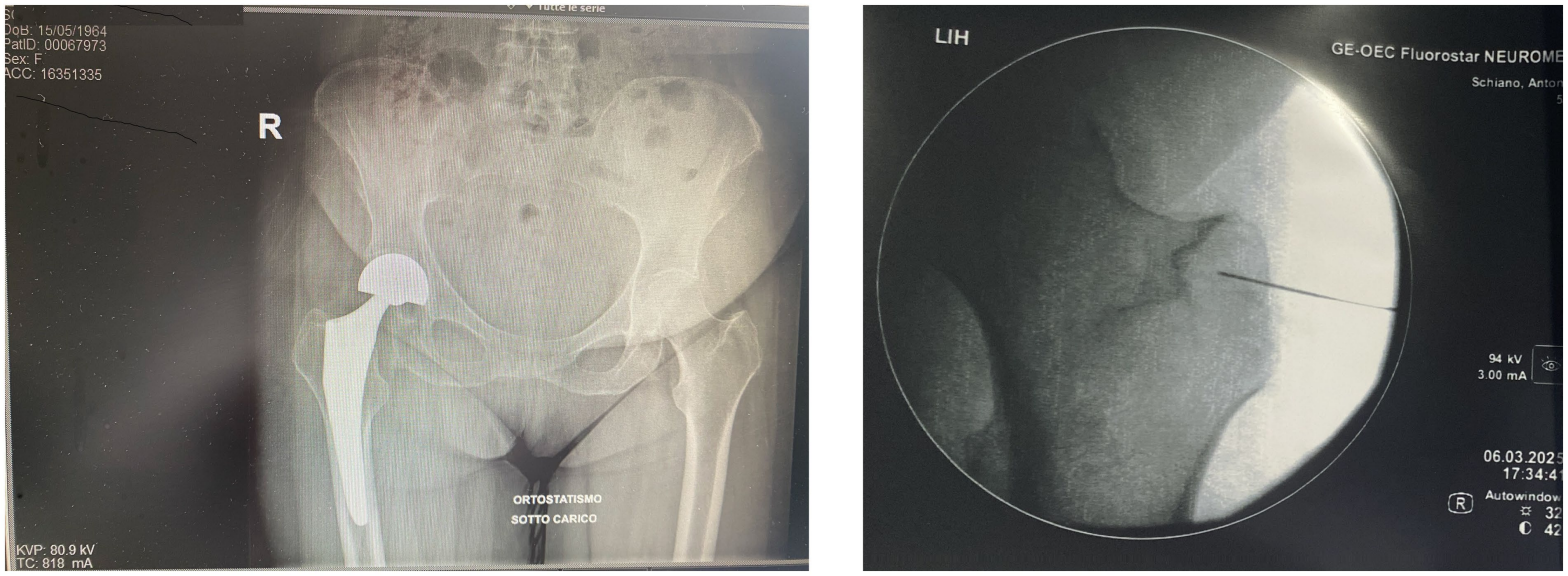
Composite image. The anteroposterior radiograph **(left)** shows our patient’s right total hip arthroplasty in a standing, weight-bearing position, with proper alignment of prosthetic components and no radiographic signs of loosening or mechanical complications. The intraoperative fluoroscopic image **(right)**, adapted from our interventional protocol, shows precise placement of pulsed radiofrequency (PRF) electrodes near the trochanteric branches of the femoral nerve. The procedure was followed by targeted PRF treatment of the articular branches of the femoral and obturator nerves under fluoroscopic guidance. PRF is used to modulate peripheral nociceptive pathways without causing structural nerve injury, representing a promising therapeutic option in patients with chronic post-arthroplasty hip pain. Reference: Chye et al. (5).

This case underscores the importance of integrating neurophysiological assessments into the rehabilitative decision-making process, especially in complex or treatment-resistant presentations of central nervous system disorders. Infact, neurophysiology offers valuable insights into functional connectivity, fatigue mechanisms, and motor control, which can inform individualized rehabilitation strategies and optimize outcomes (21, 23, 24) (Figure 1).

## Conclusion

The analysis of the clinical case presented highlights the efficacy and relevance of pulsed radiofrequency (PRF) as a targeted therapeutic option in patients affected by Spasticity-Plus Syndrome (SPS) secondary to multiple sclerosis. The combination of selective diagnostic blocks and subsequent neuromodulatory treatment using PRF on the articular nerves resulted in a significant reduction in both pain and irritative motor symptoms, suggesting a direct role for peripheral modulation in the management of dysfunctional spinal circuits involved in SPS. Although pulsed radiofrequency (PRF) is a minimally invasive technique, it is widely adopted and well-tolerated, particularly when applied to periskeletal targets. In these regions, the anatomical distance from critical neurovascular structures ensures a low risk of infection or neural injury, and the procedures are generally straightforward to perform under imaging guidance. By contrast, non- invasive neuromodulation techniques such as repetitive transcranial magnetic stimulation (rTMS) and transcranial direct current stimulation (tDCS) offer an excellent safety profile, with virtually no procedural risks. While both approaches are valid, it is worth noting that rTMS received formal clinical endorsement for chronic pain management only last years, while RF has been integrated into interventional pain protocols for over two decades, and remains more commonly employed in routine clinical settings, particularly in Italy. The improvement obtained proved to be superior to that achieved with traditional pharmacological treatments (25), including the use of oral antispastic agents, opioids, and botulinum toxin, suggesting that PRF may represent not only a salvage strategy in refractory cases but also an integral component of the multimodal approach to complex spasticity. The efficacy of PRF is based on its ability to selectively modulate the excitability of sensory branches of articular nerves, reducing peripheral nociceptive input that fuels hyperexcitable spinal reflexes, with beneficial effects on spasticity, spasms, and clonus. From a broader perspective, PRF could represent a therapeutic option indicated for all those patients with MS and SPS who present with mixed articular pain, localized segmental hypertonia, and muscle spasms refractory to first-line therapies. The use of pulsed radiofrequency (PRF) in this case suggests that it may represent a potential complementary strategy in the treatment of Spasticity-Plus Syndrome (SPS), especially in cases with limited response to pharmacological approaches. While clinical improvement was notable, these findings must be interpreted within the context of a single case and require further validation in controlled studies before drawing comparisons with conventional treatments However, its safety profile, repeatability, and absence of permanent neural injury make it a technique compatible with the long-term management needs of chronic neurological patients, placing it within a personalized, dynamic, and patient-centered treatment framework.

## Data Availability

The raw data supporting the conclusions of this article will be made available by the authors, without undue reservation.
